# *ERCC1* and *ERCC2* Variants Predict Survival in Gastric Cancer Patients

**DOI:** 10.1371/journal.pone.0071994

**Published:** 2013-09-02

**Authors:** Yangkai Li, Zhensheng Liu, Hongliang Liu, Li-E Wang, Dongfeng Tan, Jaffer A. Ajani, Qing-Yi Wei

**Affiliations:** 1 Department of Epidemiology, the University of Texas MD Anderson Cancer Center, Houston, Texas, United States of America; 2 Department of Pathology, the University of Texas MD Anderson Cancer Center, Houston, Texas, United States of America; 3 Department of Gastrointestinal Medical Oncology, the University of Texas MD Anderson Cancer Center, Houston, Texas, United States of America; Tongji Medical College, Huazhong University of Science and Technology, China

## Abstract

**Purpose:**

*ERCC1* and *ERCC2* play critical roles in the nucleotide excision repair pathway that effectively repairs DNA damage induced by chemotherapeutic agents. Therefore, functional single nucleotide polymorphisms (SNPs) in these genes could have an impact on clinical outcomes in cancer patients who received chemotherapy. However, few studies have simultaneously investigated the roles of *ERCC1* and *ERCC2* SNPs in clinical outcomes in gastric cancer patients.

**Experimental Design:**

We genotyped by the TaqMan assay three common, potentially functional *ERCC1* (rs3212986) and *ERCC2* SNPs (rs13181 and rs1799793) in 360 gastric cancer patients. We used both Kaplan-Meier tests and Cox proportional hazards models to evaluate the effects of *ERCC1* and *ERCC2* genotypes and haplotypes on clinical outcomes.

**Results:**

We found that, compared with *ERCC2* rs1799793 GG+AG genotypes, the homozygous variant AA genotype was associated with significantly poorer overall survival (OS) (AA vs. GG+AG, log-rank *P* = 0.012) and significantly higher risk of death (AA vs. GG+AG, Adjusted hazards ratio [HR] 2.13; 95% CI, 1.28 to 3.56; *P* = 0.004). In combined analyses, patients with any one of the three unfavorable genotypes (i.e. *ERCC1* rs3212986 TT, *ERCC2* rs13181 GG and rs1799793 AA) had statistically significant hazards of poor prognosis (Adjusted HR, 1.54; 95% CI, 1.06 to 2.25; *P* = 0.025), compared with those without any unfavorable genotypes. Furthermore, the haplotype A-G-G (rs1799793/rs13181/rs3212986) had a significant impact on OS (Adjusted HR, 1.57; 95% CI, 1.11 to 2.21; *P* = 0.011), compared with the common haplotype G-T-G.

**Conclusion:**

*ERCC1* and *ERCC2* functional SNPs may jointly affect OS in Caucasian gastric cancer patients. Additional large prospective studies are essential to confirm our findings.

## Introduction

Gastric cancer is the second leading cause of cancer deaths, ranking the fourth most common cancer in the world, with approximately 989,600 new cases and 738,000 deaths every year [1]. In the United States, there were estimated 21,600 new cases and 10990 deaths in 2013 [2]. For gastric cancer patients, the TNM (tumor/nodule/metastasis) stage is the most acceptable measurement for evaluating effects of therapies and prognosis; however, it is not uncommon that patients with the same tumor stage and treatment may have various outcomes. Therefore, for surgeons and oncologists, it would be helpful to improve the accuracy in predicting clinical outcome by identifying genetic makers that could facilitate individualized anticancer therapy, post-operational adjunctive treatment and follow-up strategies. In recent decades, studies have found that genetic variations play roles in the development and progression of gastric cancer. Individual differences or variations in response to chemotherapies or radiotherapies are likely due to their different genetic make-ups, leading to different clinical outcomes. For example, single nucleotide polymorphisms (SNPs) are the most commonly investigated genetic variation that may influence patients' clinical outcomes [3], [4], [5].

DNA repair system plays a vital role in maintaining the stability of cellular functions and genomic integrity through the reversal of the damaged DNA induced by various endogenous and/or exogenous factors, including therapeutic agents; therefore, the host DNA repair capacity may contribute to cancer patient outcomes [6], [7]. Among the well-known DNA repair pathways, the nucleotide excision repair (NER) pathway is an important mechanism that maintains genomic integrity by removing DNA bulky lesions or interstrand adducts induced by exogenous and/or endogenous factors [8], [9]. The excision-repair cross-complementing complementation group 1 (ERCC1) and excision-repair cross-complementing complementation group 2/xeroderma pigmentosum group D (ERCC2/XPD) proteins are the two major components of the NER process. ERCC1, encoded by the gene located at chromosome 19q13.32, interacts with XPA/XPF and other NER proteins, guiding the 5′ incision activity in the NER pathway [10], whereas ERCC2, encoded by the gene located at chromosome 19q13.3, is an ATP-dependent helicase that mediates DNA unwinding for the initiation of NER [11].

To date, there are 36 coding SNPs in *ERCC1* and 115 coding SNPs in *ERCC2* have been reported (http://www.ncbi.nlm.nih.gov/snp/). In those coding SNPs, the *ERCC2* rs13181 and rs1799793 are the only two nonsynonymous SNPs with minor allele frequency >5%, and these two SNPs have been shown to have an effect on DNA repair capacity phenotype, possibly by altering the amino acid sequence of the protein [12], [13]. In addition, another common regulatory SNP at the 3′ untranslated region of *ERCC1* rs3212986 was reported to be correlated with the DNA repair capacity phenotype [14] and to have some effect on *ERCC1* mRNA expression [15]. Some studies have suggested that low *ERCC1* expression is associated with increased chemotherapeutic sensitivity and thus considered a predictive marker for patients with non-small cell lung cancer (NSCLC) treated with platinum-based chemotherapy [16], while other studies indicated that *ERCC2* SNPs were associated with poorer survival in patients with NSCLC in Chinese populations [17]. Furthermore, *ERCC2* rs13181 and rs1799793 SNPs have been shown to be prognostic predictors for patients with osteosarcoma [18], colorectal cancer [19], and oral cancer [20], but to date, few studies have investigated prognostic importance of these SNPs in gastric cancer patients. For example, only a small study showed that *ERCC2 Lys^751^Gln* (rs13181) SNP might be a predictive maker for outcomes in Hispanic patients with stage III/IV gastric cancer [21].

Although both *ERCC1* and *ERCC2* are located on chromosome 19 and participate in the NER activity, few studies have simultaneously investigated the effects of *ERCC1* and *ERCC2* variants on gastric clinical outcomes, as summarized in our previous review in a meta-analysis [22]. Therefore, we hypothesized that functional SNPs and haplotypes of these two genes may have an impact on gastric cancer prognosis. In the present study, we assessed associations of *ERCC1* (rs3213986) and *ERCC2* (rs13181, and rs1799793) SNPs with survival in gastric cancer patients.

## Materials and Methods

### Ethics statement

This study protocol was approved by The University of Texas M. D. Anderson Cancer Center Institutional Review Board (IRB), and all patients provided their informed consent using the IRB-approved informed consent form.

### Patient recruitment and follow-up

This study included 360 patients who were accrued from those with newly diagnosed and histologically confirmed gastric cancer, regardless of age, sex, or tumor stage, at The University of Texas MD Anderson Cancer Center, Houston, Texas between February, 1990 and April, 2012. Those patients who were not newly diagnosed or treated elsewhere before coming to MD Anderson Cancer Center were excluded from this study. We interviewed each eligible participant to obtain data on tobacco smoking and alcohol use. Those who had smoked less than 100 cigarettes in their lifetime were considered as “never smokers”, and all others were considered as “ever smokers”. Similarly, subjects who had drunk alcoholic beverages at least once a week for more than 1 year previously were defined as “ever drinkers”, and all others were defined as “never drinkers”. Each patient donated a one-time 10 ml of the whole blood drawn into a heparinized tube for genetic testing. All the patients included in the present study had available blood samples for genotyping and follow-up data for assessing the outcomes.

### Genotyping

Genomic DNA was isolated from the buffy coat fraction of the blood sample, using a Blood Mini Kit (Qiagen, Valencia, CA) according to the manufacturer's instructions. DNA concentrations were determined by spectrophotometric measurement of absorbance at 260 nm and the purities were calculated by A260/A280 ratio using Nanodrop ND-1000 spectrophotometer (Thermo Scientific, Rockford, IL). Genotypes of the three selected SNPs (*ERCC1* rs3213986 G>T, *ERCC2* rs13181 T>G and rs1799793 G>A) were performed using the TaqMan methodology in 384-well plates and read with the Sequence Detection Software on the ABI Prism 7900 instrument according to the manufacturer's instructions (Applied Biosystems, Foster City, CA). The primers used for rs3213986 G>T, rs13181 T>G, and rs1799793 G>A were CACAGGCCGGGACAAGAAGCGGAAG[A/C]AGCAGCAGCAGCAGCCTGTGTAGTC, TGCTGAGCAATCTGCTCTATCCTCT[G/T]CAGCGTCTCCTCTGATTCTAGCTGC, and CGGGGCTCACCCTGCAGCACTTCGT[C/T]GGGCAGCACGGGGTTGGCCAGGTGG, respectively. Each 384-well plate had four negative controls, one positive control and eight repeat samples. PCR reactions were done under the following conditions: 50°C for 2 min, 95°C for 10 min, and then 95°C for 15 s and 60°C for 1 min for 40 cycles. For all genotypes, the assay success rate was >99% and the results of repeated samples were 100% concordant.

### Statistical Analysis

The goodness-of-fit χ^2^ test was applied to calculate the Hardy-Weinberg equilibrium of genotype distribution. The Kaplan-Meier method was used to visualize overall survival (OS) by three genotype groups. The OS time was calculated from the date of registration at M.D. Anderson to the date of last contact or death, and patients who were still alive at the last contact were considered censored in the analysis. The median survival time (MST) was calculated, and the log-rank test was applied to test for equality of the survival distributions. Univariate and multivariate Cox proportional hazards models were performed to calculate hazards ratios (HRs) and 95% confidence intervals (95% CIs) of each genotype to estimate its effect on survival with or without adjustment for confounding factors. Haplotypes were inferred by using the SAS PROC HAPLOTYPE process, and associations between haplotypes and OS were determined by the recessive genetic model following the xeroderma pigmentosum (XP) genetic model, as we used previously [23]. All statistical tests were two-sided, with a *P* value of 0.05 considered significant and all analyses were performed using SAS software version 9.2 (SAS Institute, Cary, NC). We also calculated the false-positive report probability (FPRP) to detect the false-positive association findings. For all the significant results, we assigned a prior probability of 0.1 to detect a HR of 2.0 for an association with genotypes and alleles of each SNP. The statistical power was also calculated using the online available PS software Version 3.0 [24], [25]. Only the significant results with statistical power >0.8 were and FPRP value <0.2 considered noteworthy association.

## Results

### Patient characteristics and clinical features

Demographic and clinical characteristics of the patients are presented in Table 1. The ages of patients were between 19 and 89 years at diagnosis with a median age of 60 years and a mean age of 59.6±12.4 years. Among the 360 patients, there were more men (221, 61.39%) than women (139, 38.61%), consisting of 224 (62.22%) non-Hispanic whites, 136 (37.78%) other ethnicities (Hispanic, Black and Asian), 191 (53.06%) ever smokers, and 188 (52.22%) ever alcohol consumers. At the last follow-up (September, 2012), 178 (49.44%) died, with a MST of 16.02 months. Of all the patients, 136 (37.78%) presented with stages I–II and 224 (62.22%) with stages III–IV. The distribution of histological types included 169 (46.94%) intestinal carcinomas, 148 (41.11%) signet ring carcinomas and 43 (11.94%) other types. The distribution of histological grades included 113 (31.39%) moderate/moderate-poor differentiation cases and 247 (68.61%) poor differentiation cases. For the treatment category, 136 (37.78%) patients had surgical treatment, 293 (81.39%) patients took chemotherapy, and 114 (31.67%) patients received radiotherapy (112 patients had both chemotherapy and radiotherapy). When all of these variables were included in a Cox proportional hazards regression model for adjustment to calculate hazards ratios (HRs), undergoing surgery (HR, 3.51; 95% CI, 2.37 to 5.20; *P*<0.001), having stage III–IV diseases (HR, 2.54; 95% CI, 1.74 to 3.69; *P*<0.001), and receiving radiotherapy (HR, 1.57; 95% CI, 1.11 to 2.21; *P* = 0.011) remained statistically significant prognostic indicators (log-rank *P*<0.05; Table 1). Although the crude *P* was 0.004 in the receiving chemotherapy group, the adjusted HR was not statistically significant (*P* = 0.902).

**Table 1:** Associations between characteristics of patients with gastric cancer and their overall survival.

			Crude			Adjusted		
Parameter	Patient No. (%)	Median Survival Time (months)	HR*	95% CI	*P* **	HR	95% CI	*P* †
Age, years								
<60	174 (48.33)	13.8	1.00			1.00		
≥60	186 (51.67)	17.1	0.84	0.62–1.12	0.233	1.01	0.74–1.37	0.975
Sex								
Male	221 (61.39)	17.0	1.00			1.00		
Female	139 (38.61)	13.7	1.27	0.92–1.74	0.142	1.02	0.71–1.47	0.919
Race								
White	224 (62.22)	17.2	1.00			1.00		
Other	136 (37.78)	13.5	0.81	0.59–1.12	0.203	0.80	0.57–1.11	0.183
Smoke								
Ever	191 (53.06)	17.2	1.00			1.00		
Never	169 (46.94)	13.5	1.02	0.76–1.37	0.907	1.27	0.89–1.81	0.181
Alcohol								
Ever	188 (52.22)	16.3	1.00			1.00		
Never	172 (47.78)	15.7	0.81	0.61–1.10	0.177	0.76	0.53–1.07	0.117
Histological Type								
Intestinal	169 (46.94)	17.3	1.00			1.00		
Signet ring	148 (41.11)	16.9	0.85	0.62–1.17	0.319	0.89	0.64–1.24	0.479
Other	43 (11.94)	10.4	1.14	0.70–1.87	0.598	1.14	0.69–1.90	0.612
Histological Grade								
Moderate/Moderate-Poor	113 (31.39)	17.2	1.00			1.00		
Poor	247 (68.61)	14.0	1.02	0.75–1.40	0.898	1.09	0.79–1.52	0.589
Stage								
I/II	136 (37.78)	22.1	1.00			1.00		
III/IV	224 (62.22)	12.0	**3.12**	**2.21–4.42**	**<0.001**	**2.54**	**1.74–3.69**	**<0.001**
Operation								
Yes	136 (37.78)	30.1	1.00			1.00		
No	224 (62.22)	11.7	**4.04**	**2.84–5.77**	**<0.001**	**3.51**	**2.37–5.20**	**<0.001**
Chemotherapy								
Yes	293 (81.39)	14.3	1.00			1.00		
No	67 (18.61)	21.1	**0.53**	**0.34–0.81**	**0.004**	1.03	0.63–1.70	0.902
Radiotherapy								
Yes	114 (31.67)	22.6	1.00			1.00		
No	246 (68.33)	12.4	**1.62**	**1.18–2.23**	**0.003**	**1.57**	**1.11–2.21**	**0.011**

* HR = hazards ratio.

** *P* values were calculated by Cox proportional model using univariate analysis.

† *P* values were calculated by Cox proportional model using multivariate analysis.

(Numbers in **bold** represent statistically significant findings).

### Association between *ERCC1* and *ERCC2* SNPs and OS

**Figure 1:**
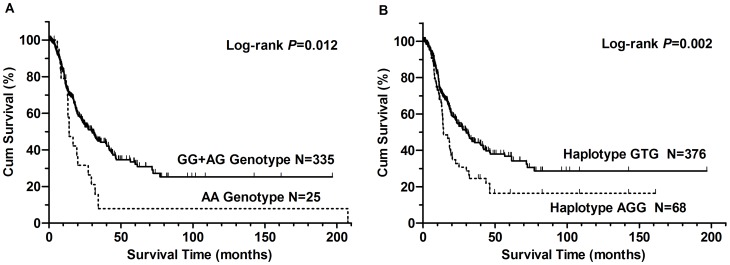
Kaplan-Meier curve of overall survival according to (A) *ERCC2* rs1799793 polymorphism and (B) *ERCC1*-*ERCC2* haplotype.

We used the Kaplan-Meier method and the Cox model to evaluate the effects of *ERCC1* and *ERCC2* SNPs on patients' OS. Table 2 shows genotype distributions of the *ERCC1* rs3213986 and *ERCC2* rs13181 and rs1799793 SNPs as well as their associations with OS. The observed genotypes in the patients agreed with those estimated from the Hardy-Weinberg equilibrium (*P* = 0.466 for rs1799793, *P* = 0.820 for rs13181, and *P* = 0.789 for rs3213986). In all patients, the *ERCC2* rs1799793 G>A SNP showed a statistically significant association with OS, and the MST for patients with AA genotype was shorter than that in those with the common GG genotype (13.2 months vs. 15.8 months; log-rank *P* = 0.012). In the Cox analysis, patients with the variant AG/AA genotypes exhibited significantly increased hazards of death in univariate models, compared with those who had the GG genotype (HR, 1.46; 95% CI, 1.07 to 2.00 and *P* = 0.017 for AG, and HR, 2.23; 95% CI, 1.33 to 3.75 and *P* = 0.002 for AA, respectively). After adjustment for sex, age, smoking status, alcohol use, tumor stage, histological grade, histological type and treatment in a multivariate Cox model, the AA genotype remained had statistically significant impact on OS, compared with rs1799793 GG genotype (HR, 2.18; 95% CI, 1.26 to 3.78 and *P* = 0.006) or GG+AG genotype (HR, 2.13; 95% CI, 1.28 to 3.56 and *P* = 0.004) (Figure 1A). For the other two *ERCC2* rs13181 and *ERCC1* rs3212986 SNPs, although both SNPs showed associations with a tendency of increased HR with variant genotypes, there was no statistically significant association with OS under a recessive genetic model (Table 2).

**Table 2:** Univariate and multivariate analyses of associations between *ERCC1* genotype, *ERCC2* genotypes, combined *ERCC1* and *ERCC2* variants and overall survival in gastric cancer patients.

Genotypes	Patient No.		Median Survival Time (months)	Log-rank *P*	Crude			Adjusted		
	Dead (%)	Alive (%)			HR	95% CI	*P* *	HR	95% CI	*P* **
*ERCC1* rs3212986 G>T										
GG	87 (24.17)	94 (26.11)	15.3		1.00			1.00		
GT	75 (20.83)	75 (20.83)	16.9	0.831	1.03	0.76–1.41	0.836	1.00	0.72–1.39	0.991
TT	16 (4.44)	13 (3.61)	17.2	0.543	1.22	0.71–2.08	0.469	1.22	0.71–2.12	0.475
Recessive model										
GG+GT	162 (45.00)	169 (46.94)	15.7		1.00			1.00		
TT	16 (4.44)	13 (3.61)	17.2	0.487	1.20	0.72–2.01	0.487	1.22	0.72–2.08	0.461
*ERCC2* rs13181 T>G										
TT	77 (21.39)	95 (26.39)	16.5		1.00			1.00		
GT	83 (23.06)	72 (20.00)	16.8	0.208	1.22	0.89–1.67	0.211	1.04	0.75–1.45	0.799
GG	18 (5.00)	15 (4.17)	13.2	0.139	1.47	0.88–2.46	0.139	1.28	0.75–2.18	0.368
Recessive model										
TT+GT	160 (44.44)	167 (46.39)	16.8		1.00			1.00		
GG	18 (5.00)	15 (4.17)	13.2	0.242	1.34	0.82–2.18	0.243	1.25	0.76–2.06	0.387
*ERCC2* rs1799793 G>A										
GG	73 (20.28)	112 (31.11)	15.8		1.00			1.00		
AG	86 (23.89)	64 (17.78)	17.1	**0.017**	**1.46**	**1.07–2.00**	**0.017**	1.04	0.74–1.47	0.822
AA	19 (5.28)	6 (1.67)	13.2	**0.002**	**2.23**	**1.33–3.75**	**0.002**	**2.18**	**1.26–3.78**	**0.006**
Recessive model										
GG+AG	159 (44.17)	176 (48.89)	16.8		1.00			1.00		
AA	19 (5.28)	6 (1.67)	13.2	**0.012**	**1.85**	**1.13–3.02**	**0.014**	**2.13**	**1.28–3.56**	**0.004**
No. of variant genotypes†										
0	140 (38.89)	157 (43.61)	16.2		1.00			1.00		
1 to 3	38 (10.56)	25 (6.94)	14.1	0.131	1.32	0.92–1.90	0.132	**1.54**	**1.06–2.25**	**0.025**

* *P* values were calculated by Cox proportional hazards model using univariate analysis.

** *P* values were adjusted for age, sex, race, smoking status, alcohol use, stage, histological grade, histological type and treatment in a Cox model.

† The variant homozygous genotypes were *ERCC1* rs3212986 TT, *ERCC2* rs13181 GG and rs1799793 AA.

(Numbers in **bold** represent statistically significant findings).

### Survival of gastric cancer patients by combined genetic risk factors

To assess the joint effect of the three SNPs on patient prognosis, we combined three variant homozygous genotypes (i.e., *ERCC1* rs3212986 TT, *ERCC2* rs13181 GG, and rs1799793 AA). Of all the patients, 63 patients had at least one variant homozygote genotype, and these patients had statistically significant hazards of poor prognosis (adjusted HR, 1.54; 95% CI, 1.06–2.25; *P* = 0.025), compared with those without any (Table 2).

### Association between *ERCC1* and *ERCC2* haplotypes and OS

Because these three SNPs are all on chromosome 19, we further explored their haplotypes and evaluated their combined effect on gastric cancer survivals. Overall, there were eight haplotypes, of which five had frequencies >5%, and other three less common haplotypes were combined into one group. The five most common haplotypes in these patients were rs1799793/rs13181/rs3212986 G-T-G, A-G-T, G-T-T, A-G-G, and G-G-G with the frequency of 52.2%, 13.9%, 12.6%, 9.4%, and 6.8%, respectively. Compared with the most common G-T-G haplotype, the A-G-G haplotype had a significant impact on OS (adjusted HR, 1.57; 95% CI, 1.11 to 2.21; *P* = 0.011) (Table 3) (Figure 1B).

**Table 3:** Univariate and multivariate analyses of associations between *ERCC1* and *ERCC2* haplotypes and overall survival in gastric cancer patients.

Haplotype*	No. (%) n = 720	Crude			Adjusted		
		HR	95% CI	*P*	HR	95% CI	*P* **
G-T-G	376(52.22)	1.00			1.00		
A-G-T	100(13.89)	1.23	0.90–1.68	0.188	1.02	0.74–1.40	0.920
G-T-T	91(12.64)	0.95	0.68–1.34	0.771	1.20	0.84–1.71	0.311
A-G-G	68(9.44)	**1.66**	**1.19–2.30**	**0.003**	**1.57**	**1.11–2.21**	**0.011**
G-G-G	49(6.81)	0.84	0.53–1.34	0.465	0.92	0.57–1.48	0.719
Other	36(5.00)	1.18	0.78–1.78	0.436	1.10	0.71–1.69	0.675

* Haplotype was composed in the order of rs1799793, rs13181, rs3213986; G-T-G was the most common haplotype.

** *P* values were adjusted for age, sex, race, smoking status, alcohol use, stage, histological grade, histological type and treatment in a Cox model.

(Numbers in **bold** represent statistically significant findings).

### Stratification analysis between the recessive genotypes and overall survival

We performed stratified analysis to investigate whether the SNPs and their combined genotypes in a recessive model on survival were modified by some important clinicopathological factors in Table 1. We found that the *ERCC2* rs1799793 AA genotype had a higher death risk in those patients who were older (≥60 years), male, non-Hispanic white, having smoking history, having poor differential, intestinal type of tumors, and having any treatment (operation, chemotherapy or radiotherapy). We also observed statistically significant poor prognosis in male patients with the *ERCC2* rs13181 GG genotype, and non-smoking patients with the *ERCC1* re3212986 TT genotype (Table 4). The combined analyses showed that patients with any of the three variant homozygote genotypes would suffer a higher risk of death, particularly in those who were older (≥60 years), male, alcohol users, non-Hispanic white, having poor differential, intestinal type of tumors with stages I/II, and having chemotherapy (Table 4).

**Table 4:** Stratification analysis for associations between *ERCC1* and *ERCC2* SNPs and overall survival in gastric cancer patients.

	*ERCC2* rs1799793 G>A					*ERCC2* rs13181 T>G					*ERCC1* rs3212986 G>T					Combined genotypes†				
Parameter	GG+AG	AA	AdjustedHR	(95% CI)	*P* *	TT+GT	GG	Adjusted HR	(95% CI)	*P* *	GG+GT	TT	Adjusted HR	(95% CI)	*P* *	0	1–3	Adjusted HR	(95% CI)	*P* *
Age, years																				
<60	166	8	1.17	0.44–3.06	0.756	159	15	0.85	0.38–1.87	0.684	160	14	1.27	0.61–2.64	0.524	145	29	1.24	0.71–2.19	0.451
≥60	169	17	**3.12**	**1.59–6.12**	**0.001**	168	18	1.82	0.91–3.62	0.090	171	15	1.17	0.53–2.57	0.705	152	34	**1.91**	**1.12–3.27**	**0.018**
Sex																				
Male	206	15	**2.21**	**1.14–4.29**	**0.018**	200	21	**1.83**	**1.01–3.33**	**0.047**	205	16	0.99	0.50–1.95	0.983	185	36	**1.78**	**1.11–2.88**	**0.018**
Female	129	10	2.12	0.84–5.32	0.111	127	12	0.67	0.23–1.94	0.460	126	13	1.83	0.73–4.56	0.198	112	27	1.30	0.67–2.53	0.436
Smoke																				
Ever	175	16	**2.23**	**1.16–4.29**	**0.017**	170	21	1.30	0.70–2.40	0.411	175	16	0.70	0.33–1.48	0.352	154	37	1.36	0.82–2.24	0.237
Never	160	9	2.14	0.87–5.28	0.099	157	12	0.98	0.38–2.53	0.969	156	13	**2.66**	**1.11–6.37**	**0.028**	143	26	1.74	0.94–3.22	0.079
Alcohol																				
Ever	178	10	2.09	0.99–4.43	0.054	173	15	1.88	0.96–3.66	0.066	173	15	1.23	0.63–2.37	0.546	159	29	**1.87**	**1.10–3.16**	**0.021**
Never	157	15	1.97	0.94–4.11	0.072	154	18	0.79	0.35–1.77	0.570	158	14	1.01	0.40–2.59	0.981	138	34	1.22	0.69–2.15	0.503
Race																				
White	202	22	**2.14**	**1.23–3.72**	**0.007**	196	28	1.62	0.95–2.75	0.077	212	12	1.01	0.50–2.03	0.989	183	41	**1.76**	**1.12–2.74**	**0.014**
Other	133	3	2.95	0.62–14.04	0.173	131	5	0.40	0.05–3.13	0.385	119	17	2.04	0.85–4.92	0.113	114	22	1.43	0.65–3.13	0.369
Histological Grade																				
Moderate/Moderate-Poor	107	6	1.60	0.37–6.91	0.530	103	10	1.37	0.48–3.88	0.554	110	3	0.43	0.06–3.31	0.419	100	13	1.00	0.38–2.62	0.996
Poor	228	19	**2.11**	**1.19–3.75**	**0.011**	224	23	1.30	0.72–2.37	0.387	221	26	1.36	0.78–2.40	0.282	197	50	**1.67**	**1.08–2.58**	**0.021**
Histological Type																				
Intestinal	155	14	**2.46**	**1.29–4.68**	**0.006**	155	14	1.69	0.85–3.36	0.136	158	11	1.12	0.50–2.52	0.784	140	29	**1.91**	**1.15–3.20**	**0.013**
Signet ring	139	9	1.86	0.62–5.58	0.271	134	14	0.78	0.29–2.07	0.615	131	17	1.28	0.58–2.81	0.539	120	28	1.13	0.57–2.24	0.736
Other	41	2	0.81	0.08–7.93	0.858	38	5	0.73	0.08–6.49	0.776	42	1	-	-	-	37	6	1.98	0.26–14.9	0.509
Stage																				
I/II	126	10	2.50	0.98–6.38	0.057	128	8	2.01	0.66–6.13	0.221	123	13	2.86	0.95–8.64	0.062	113	23	**2.28**	**1.07–4.86**	**0.034**
III/IV	209	15	1.72	0.90–3.31	0.104	199	25	1.15	0.65–2.05	0.623	208	16	1.03	0.53–1.97	0.942	184	40	1.38	0.88–2.18	0.163
Surgery																				
Yes	127	9	**3.17**	**1.18–8.51**	**0.022**	126	10	1.45	0.51–4.10	0.483	125	11	1.12	0.31–4.01	0.863	112	24	1.63	0.77–3.46	0.206
No	208	16	1.87	0.99–3.51	0.052	201	23	1.28	0.71–2.32	0.417	206	18	1.22	0.67–2.24	0.517	185	39	1.56	0.99–2.47	0.057
Chemotherapy																				
Yes	271	22	**2.16**	**1.25–3.73**	**0.006**	263	30	1.27	0.76–2.13	0.366	271	22	1.20	0.68–2.11	0.531	240	53	**1.57**	**1.06–2.33**	**0.026**
No	64	3	4.24	0.73–24.78	0.108	64	3	0.45	0.02–9.39	0.610	60	7	1.33	0.19–9.24	0.775	57	10	0.93	0.19–4.43	0.925
Radiotherapy																				
Yes	106	8	**7.73**	**2.82–21.18**	**<0.001**	102	12	1.90	0.77–4.68	0.162	107	7	1.07	0.32–3.63	0.910	94	20	2.07	0.94–4.55	0.069
No	229	17	1.56	0.82–2.99	0.180	225	21	1.06	0.55–2.04	0.853	224	22	1.21	0.66–2.19	0.541	203	43	1.43	0.90–2.27	0.135

* *P* values were adjusted for age, sex, race, smoking status, alcohol use, stage, histological grade, histological type and treatment in the cox model.

† The combined three variant homozygous genotypes were *ERCC1* rs3212986 TT, *ERCC2* rs13181 GG and rs1799793 AA.

(Numbers in **bold** represent statistically significant findings).

Finally, we calculated the FPRP values at different prior probability levels as well as statistical power for all significant findings (Table 5). For a prior probability of 0.1, assuming the HR for specific genotype was 2.0, the FPRP values were 0.044, 0.079, 0.087 and 0.197, with statistical power of 0.930, 0.927, 0.903 and 0.842 (The Type I error probability associated with this test of this null hypothesis is 0.05), respectively, for an association of the *ERCC2* rs1799793 AA genotype, A-G-G haplotype and 1–3 of *ERCC1* and *ERCC2* risk genotypes, respectively, suggesting that these may be noteworthy findings in all individuals, and we would be able to reject the null hypothesis that the variant and reference survival curves were equal with statistical power>0.8. In addition, most of the significant associations in stratified analyses were considered noteworthy because the probability of a false-positive result was <0.2 and statistical power >0.8 (Table 5). Nevertheless, some greater FPRP values (>0.2) were observed for some other significant associations in stratified analyses (e.g. *ERCC2* rs13181 and *ERCC1* rs3213986 variant genotypes with gastric cancer OS). For those significant associations with statistical powers <0.8, we could not reject the null hypothesis that the survival curves were equal between the variant and the reference groups, and that were corresponding to the greater FPRP values (>0.2) (Table 5), suggesting some possible bias in these positive findings that need to be validated in larger studies in the future.

**Table 5:** False-positive report probability for Association between overall survival in gastric cancer patients and the genotypes and haplotypes of *ERCC1* and *ERCC2* variants.

Genotypes/Haplotypes	HR*	(95%CI)	*P* * value	Statistical Power	Prior Probability		
					0.250	0.100	0.010
*ERCC2* rs1799793							
AA vs. GG							
All patients	2.18	1.26–3.78	0.006	0.930	0.015	**0.044**	0.334
AA vs. GG+AG							
All patients	2.13	1.28–3.56	0.004	0.927	0.028	**0.079**	0.486
Age≥60 y	3.12	1.59–6.12	0.001	0.982	0.028	**0.080**	0.488
Male	2.21	1.14–4.29	0.018	0.788	0.126	0.303	0.827
Ever smoking	2.23	1.16–4.29	0.017	0.812	0.117	0.284	0.814
White	2.14	1.23–3.72	0.007	0.883	0.051	**0.139**	0.639
Poor differentiation	2.11	1.19–3.75	0.011	0.841	0.071	**0.188**	0.718
Intestinal type	2.46	1.29–4.68	0.006	0.844	0.066	**0.174**	0.699
Surgery	3.17	1.18–8.51	0.022	0.796	0.218	0.455	0.902
Chemotherapy	2.16	1.25–3.73	0.006	0.905	0.042	**0.115**	0.589
Radiotherapy	7.73	2.82–21.18	<0.001	0.985	0.047	**0.128**	0.617
*ERCC2* rs13181							
GG vs. TT+GT							
Male	1.83	1.01–3.33	0.047	0.695	0.186	0.406	0.883
*ERCC1* rs3212986							
TT vs. GG+GT							
Never smoking	2.66	1.11–6.37	0.028	0.890	0.242	0.490	0.914
Haplotype†							
A-G-G vs. G-T-G	1.57	1.11–2.21	0.011	0.903	0.031	**0.087**	0.512
Combined genotypes							
0 variant vs. 1–3 variants							
All patients	1.54	1.06–2.25	0.025	0.842	0.075	**0.197**	0.729
Age≥60 y	1.91	1.12–3.27	0.018	0.893	0.088	0.225	0.761
Male	1.78	1.11–2.88	0.018	0.844	0.073	**0.190**	0.721
Ever alcohol using	1.87	1.10–3.16	0.021	0.831	0.094	0.237	0.774
White	1.76	1.12–2.74	0.014	0.867	0.053	**0.143**	0.648
Poor differential	1.67	1.08–2.58	0.021	0.873	0.072	**0.189**	0.720
Intestinal tumor	1.91	1.15–3.20	0.013	0.843	0.066	**0.174**	0.698
Stage I/II	2.28	1.07–4.86	0.034	0.904	0.214	0.449	0.900
Chemotherapy	1.57	1.06–2.33	0.026	0.811	0.079	0.205	0.740

* The adjusted HR and *P* value reported in Table 2–4.

† Haplotype was composed in the order of rs1799793, rs13181, rs3213986; G-T-G was the most common haplotype.

(Numbers in **bold** represent statistically significant findings.).

## Discussion

In the present study, we investigated whether functional *ERCC1* rs3213986 G>T and *ERCC2* rs13181 T>G and rs1799793 G>A SNPs have any individual or joint effects on clinical outcomes of gastric cancer patients in a North American patient population. We found that the *ERCC2* rs1799793 AA genotype contributed to a significantly shorter survival time, compared with the AG/GG genotype. After adjustment for other clinical factors, the effects of the *ERCC2* rs1799793 AA genotype, the combined effects of the three variant homozygote genotypes, and the rs1799793/rs13181/rs3212986 haplotype A-G-G all remained as significant predictors for poorer clinical outcomes in this study population. Additionally, these effects also remained in stratification analyses. Overall, patients with the *ERCC2* rs1799793 AA genotype would have higher death risk, particularly in those who were older (≥60 years), non-Hispanic white, having poor differential, intestinal type of tumors, and having chemotherapy or radiotherapy. Furthermore, patients with any of the rs1799793/rs13181/rs3212986 variant homozygote genotypes would suffer a higher risk of death, particularly in those who were male, non-Hispanic white, having poor differential and intestinal type of tumors.

It is well known that some clinicopathological characteristics, such as tumor stage and treatment, are associated with prognosis of cancer patients. However, we were interested in functional genetic variants of DNA repair genes that may modulate patients' survival in response to chemotherapy. The NER process is an important DNA repair mechanism that maintains genomic integrity, both in normal and tumor cells, and the changes in the NER capacity may either increase or reduce DNA mutation frequencies as a result of unrepaired damaged DNA, which may alter patients' response to the therapy, and thus may lead malignant progression and metastasis. ERCC1 and ERCC2 are two key rate-limiting enzymes acting in the multistep NER process. ERCC1 is involved in DNA damage recognition, and ERCC2 is a member of a nine-subunit complex human transcriptional initiation factor TFIIH with ATP-dependent helicase activity. Therefore, potentially functional *ERCC1* and *ERCC2* SNPs may affect cellular DNA repair capacity [26] and thus may be associated with various survival rates observed in cancer patients.

Previous reports on the association between *ERCC1* rs3212986 and cancer survival have been controversial. For example, Takenaka et al. demonstrated that *ERCC1* rs3213986 SNP might influence the NSCLC prognosis regardless of the *ERCC1* expression and platinum sensitivity in 122 Japanese NSCLC patients who underwent a complete resection [27]; however, KimCurran's study showed that there was lack of correlation between *ERCC1* C8092A (rs3212986) SNP and OS or efficacy/toxicity of platinum-based chemotherapy in 300 Chinese NSCLC patients treated with chemotherapy [28]. Furthermore, a recent meta-analysis showed there was no evidence to support *ERCC1* C118T (for 1187 pooled patients)/C8092A (for 625 pooled patients) SNPs as a prognostic predictor of platinum-based chemotherapy in patients with NSCLC [29]. Indeed, in the present study of 360 gastric cancer patients, we did not find any association between *ERCC1* rs3212986 G>T SNP and OS. The stratification analysis showed that only in non-smokers, the rs3212986 TT genotype was associated with a significantly higher HR (*P* = 0.028) after adjustment for clinical factors. But after calculating FPRP for this significant finding, a greater FPRP value was observed, which suggests a possible bias in the estimate may have occurred due to a relatively small sample size in the present study. There was no predicting value of rs3212986 genotypes for OS in patients who received chemotherapy or radiotherapy, particularly in non-Hispanic whites, which are the majority of all the patients.

*ERCC2* rs13181 and rs1799793 SNPs have also previously been investigated for their roles in cancer patient survival. Wu *et al.* indicated that patients with *XPD Lys^751^Gln* (rs13181) and *Asp^312^Asn* (rs1799793) variant genotypes had significantly poorer NSCLC survival in Chinese patients [17]. Previous studies have also shown that *ERCC2* rs13181 G allele and rs1799793 A allele might lead to a low NER capacity, compared with the common genotypes [30], [31], [32] and thus might influence patient outcomes. The present study showed that the *ERCC2* rs1799793 AA genotype was associated with OS in gastric cancer patients. In further stratification analyses, we found that the HRs were increased in the subgroup of older age (≥60 years), male, having smoking history, non-Hispanic white patients, intestinal tumor, having surgery therapy, chemotherapy and radiotherapy, which may indicate poorer outcomes. We also observed rs1799793 AA genotype had significant impact on OS in patients who had poorly differentiated tumor, but no significant difference was found between different tumor stages. However, the FPRP and statistical power calculation showed possible noteworthy associations with poor OS only in the subgroups of older age, non-Hispanic white, poorly differentiated tumor, intestinal type of tumor and having chemotherapy or radiotherapy. Therefore, some of our findings from the stratified analyses may be by chance, because of reduced sample size in the subgroups. These findings, once validated in larger studies, may be useful in evaluating treatment strategy based on genetic background for patients with different clinicopathological characteristics.

Previous studies have also demonstrated some conflicting data regarding the role of *ERCC2* rs13181 T>G SNP in survival of cancer patients. A study of 106 colorectal cancer cases in a North American population found that the rs13181 GG genotype was associated with increased risk of death [33], and another investigation of 72 Spanish patients with colorectal cancer showed that these patients with the rs13181 heterozygote displayed higher risk than carriers of TT or GG genotypes [34]; In contrast, Keam and colleagues did not find any influence of rs13181 genotypes on OS in a study of 73 Asian patients with gastric cancer [35], and Liu *et al.* indicated that *XPD* Lys^751^Gln (rs13181) could not be genetic determinant for prognosis of advanced NSCLC treated with platinum-based chemotherapy [36]. In the present study of 360 patients with gastric cancer, we did not observe any significant association between rs13181 genotypes and overall survival, nor in stratification analyses. The possible explanation is that the allele frequency of rs13181 SNP may vary in different ethnic groups (e.g., the rs13181 GG variant genotype has a very low frequency in Asian population), and the numbers of cases in prior studies were too small (less than 100 cases in Spanish and Asian studies), which could have introduced biases in their findings. To the best of our knowledge, the present study is the largest case-only study focusing on the rs13181 SNP in gastric cancer OS in a North American populations with a majority of Caucasians, but larger studies are needed to validate our findings.

The effect of a single SNP on death risk or clinical outcomes, if any, may be limited as one would expect, and the combined effects of several SNPs in *ERCC1* and *ERCC2* on the same chromosome may be more significant. Indeed, in the present study, we observed that patients who had at least one of the three unfavorable variant homozygous genotypes (rs1799793 AA, rs13181 GG and rs3212986 TT) had a markedly increased risk of death, compared with those with none of the unfavorable variant homozygous genotypes. In further stratification analysis, we found that the genotype-survival association remained significant in the presence of most clinicopathological risk factors. After FPRP and statistical power calculation, the significant associations were noteworthy for the subgroups of male, non-Hispanic white, poorly differentiated tumor and intestinal type of gastric cancer patients, who were carrying at least one unfavorable rs1799793/rs13181/rs3212986 variant homozygote. These findings are biologically plausible, because all these unfavorable variant homozygous genotypes may collectively have a substantial effect on the DNA repair capacity. Furthermore, a more significant association was found with the poorer survival in the haplotype analysis. Patients with the rs1799793/rs13181/rs3212986 (A-G-G) haplotype had a significantly poorer survival, compared with patients with the wild-type G-T-G haplotype. This effect remained significant after adjustment (*P* = 0.011), FPRP and statistical power calculation. The cumulative effect of the haplotype might be the result of the synergistic of each SNP [17]. Nevertheless, the A-G-T haplotype, which was composed by all rs1799793A/rs13181G/rs3212986T variant genotypes, was not association with OS in the present study. One of the possible explanations is that the *ERCC1* rs3212986 variant T genotype is not associated with OS [28], [29], but the G genotype might have synergistic effect with *ERCC2* rs1799793/rs13181 on OS, which needs to be further validated in larger studies.

The present study has some limitations. First, we were unable to explore the exact mechanism by which *ERCC1* and *ERCC2* SNPs influence gastric cancer survival. Second, though the present study included a relatively large number of gastric patients, compared with previously published studies of Caucasians, the number of cases with variant homozygous genotypes was still small, which may lead to statistical bias in the analyses, and we are planning to confirm current findings in our ongoing study of a larger study population with a longer follow-up time. Third, we used the common functional SNPs in our investigation, which did not include all representative SNPs in the entire gene. Some other rare functional SNPs, which may influence survival, may have been missed and need to be investigated in larger studies. Forth, due to the limitation of unavailable clinical data, we were not able to evaluate neither the disease-free survival nor the potential role of the SNPs by different chemotherapy/radiotherapies, and fifth, although we had significant results, there was no independent replication population readily available for the present study, which should be done in the future to validate our findings.

In summary, we found that *ERCC1* and *ERCC2* functional SNPs may independently (rs1799793) and jointly (rs1799793, rs13181 and rs3212986) affect the OS in North American gastric cancer patients. Additional large, prospective studies are essential to confirm our findings.
